# Genetic testing for mitochondrial disease: the United Kingdom best practice guidelines

**DOI:** 10.1038/s41431-022-01249-w

**Published:** 2022-12-13

**Authors:** Eleni Mavraki, Robyn Labrum, Kate Sergeant, Charlotte L. Alston, Cathy Woodward, Conrad Smith, Charlotte V. Y. Knowles, Yogen Patel, Philip Hodsdon, Jack P. Baines, Emma L. Blakely, James Polke, Robert W. Taylor, Carl Fratter

**Affiliations:** 1 NHS Highly Specialised Service for Rare Mitochondrial Disorders, Newcastle upon Tyne Hospitals NHS Foundation Trust, Newcastle upon Tyne, UK; 2 Wellcome Centre for Mitochondrial Research, Translational and Clinical Research Institute, Faculty of Medical Sciences, Newcastle University, Newcastle upon Tyne, UK; 3 Neurogenetics Unit, National Hospital for Neurology and Neurosurgery, Queen Square, London, UK; 4 Oxford Genetics Laboratories, Oxford University Hospitals NHS Foundation Trust, Oxford, UK

**Keywords:** Genetic testing, Genetic testing, Neuromuscular disease, Medical genomics

## Abstract

Primary mitochondrial disease describes a diverse group of neuro-metabolic disorders characterised by impaired oxidative phosphorylation. Diagnosis is challenging; >350 genes, both nuclear and mitochondrial DNA (mtDNA) encoded, are known to cause mitochondrial disease, leading to all possible inheritance patterns and further complicated by heteroplasmy of the multicopy mitochondrial genome. Technological advances, particularly next-generation sequencing, have driven a shift in diagnostic practice from ‘biopsy first’ to genome-wide analyses of blood and/or urine DNA. This has led to the need for a reference framework for laboratories involved in mitochondrial genetic testing to facilitate a consistent high-quality service. In the United Kingdom, consensus guidelines have been prepared by a working group of Clinical Scientists from the NHS Highly Specialised Service followed by national laboratory consultation. These guidelines summarise current recommended technologies and methodologies for the analysis of mtDNA and nuclear-encoded genes in patients with suspected mitochondrial disease. Genetic testing strategies for diagnosis, family testing and reproductive options including prenatal diagnosis are outlined. Importantly, recommendations for the minimum levels of mtDNA testing for the most common referral reasons are included, as well as guidance on appropriate referrals and information on the minimal appropriate gene content of panels when analysing nuclear mitochondrial genes. Finally, variant interpretation and recommendations for reporting of results are discussed, focussing particularly on the challenges of interpreting and reporting mtDNA variants.

## Introduction

Primary mitochondrial disease describes a diverse group of neuro-metabolic disorders characterised by impaired oxidative phosphorylation. Mitochondrial disorders are clinically heterogeneous affecting isolated or multiple organ systems, may present at any age and are associated with significant morbidity and mortality [1]. Mitochondrial disease prevalence is estimated to be approximately 12.5 per 100,000 in adults [2] and approximately 4.7 per 100,000 in children [3]. However, the frequency of pathogenic mtDNA variants in the general population is estimated to be higher, with approximately 1 in 250 healthy individuals carrying a pathogenic mtDNA variant at low levels [4].

Mitochondria are double-membrane organelles found in all nucleated human cells whose primary function is the generation of adenosine triphosphate (ATP) via oxidative phosphorylation (OXPHOS) [5], but are also essential in other cellular processes including signalling pathways and apoptosis [6, 7]. Multiple mitochondria are found within cells and are generally present in higher numbers in energy-demanding cells. Human mitochondrial DNA (mtDNA) is a circular genome, consisting of 16,569 base pairs, that contains a noncoding D‐loop region regulating mtDNA replication and transcription, 13 protein-coding genes encoding OXPHOS subunits and 22 tRNA genes and 2 rRNA genes required for the transcription and translation of the mtDNA–encoded proteins [8]. Most mitochondrial proteins required for OXPHOS processes as well as the maintenance, replication and transcription of the mitochondrial genome are encoded by nuclear genes, with the mitoproteome currently estimated to comprise a total of 1136 mitochondrial proteins [9].

mtDNA is a multicopy genome, and variants can be found in all copies (homoplasmy) or there may be a coexistence of more than one mtDNA genotype within the same cell (heteroplasmy). Due to the random segregation of mitochondria during cell division, the proportion of mtDNA with a particular variant can vary between tissues of the same individual or within the same tissue over time. mtDNA exhibits strict maternal inheritance [10, 11] which is complicated by the mitochondrial genetic bottleneck that occurs during embryonic development of the female germline and can result in a rapid shift in mtDNA variant levels within one generation. Clinical manifestations associated with a heteroplasmic pathogenic variant usually become apparent when the proportion of mutant mtDNA exceeds a critical threshold within a given tissue (threshold effect), and this threshold differs depending upon the mtDNA variant [1].

Mitochondrial disease may result from defects in either the nuclear or mitochondrial genome and therefore is associated with all possible inheritance patterns: maternal, autosomal dominant, autosomal recessive, X-linked and de novo. Primary mtDNA pathogenic variants include single-nucleotide variants (SNVs) and genomic rearrangements, most commonly involving single large-scale deletions. Multiple mtDNA deletions and decreased mtDNA copy number leading to mtDNA depletion can both occur secondary to a primary nuclear gene defect. Testing an appropriate tissue for accurate genetic diagnosis is essential as some pathogenic variants progressively decline in blood with increasing age (e.g., *MT-TL1* m.3243A>G (NC_012920.1)) [12] and others can be restricted to skeletal muscle (e.g., *MT-TP* m.15975T>C, m.16002T>C, m.15998A>T (NC_012920.1) [13] and large-scale mtDNA deletions [14]).

Accurate genetic testing and establishing a genetic diagnosis are important for patients and their families, providing information about prognosis and treatment, in addition to genetic counselling and access to reproductive options. There is a need for published guidelines for genetic testing for mitochondrial disease, to help the European and international mitochondrial diagnostic community achieve a consistent high-quality service. These recommendations represent a consensus reference framework collated by three, highly specialised, UK National Health Service (NHS) laboratories that work closely together to offer diagnostic testing for the whole country [15, 16]. This unique collaborative approach of sharing experience and expertise across three centres has facilitated a comprehensive review and analysis of diagnostic practices, the results of which are presented here as best practice guidelines, highlighting fundamental aspects of the laboratory diagnosis of mitochondrial disease, strategies for diagnostic testing, family testing and reproductive options for both mtDNA and nuclear genetic variants, in addition to discussing testing methods, interpretation and reporting.

## Methods

The recommendations outlined in this document were reached using a stepwise approach: a working group of Clinical Scientists from the United Kingdom NHS Highly Specialised Services for Rare Mitochondrial Disorders revised and updated existing UK guidance collated in 2008, taking into account recent developments in mitochondrial genomics and genomic testing in the UK; this was then followed by UK-wide laboratory consultation and ratification through the Association for Clinical Genomic Science (ACGS) [17]. This initial draft was subsequently condensed for publication with some modifications for an international readership.

## Reason for referral and genetic testing strategies

Mitochondrial disorders are challenging to diagnose due to clinical and genetic heterogeneity and limited genotype-phenotype correlations. Hence, effective genetic diagnosis often necessitates a multidisciplinary approach. The advent of next-generation sequencing technologies (NGS) has driven a shift in diagnostic practices from a ‘biopsy first’ approach, where first-line biochemical and histochemical investigations of patient’s muscle biopsy were used to direct subsequent molecular analyses, to genome-wide strategies which can be employed using blood DNA and expedite the diagnosis of many cases, especially those associated with nuclear gene defects [18, 19]. The systematic analysis of the entire mitochondrial genome by NGS increases sensitivity compared to targeted common pathogenic mtDNA variant testing and facilitates accurate heteroplasmy assessment [20], although it is important to note that more comprehensive testing also increases the likelihood of detecting variant(s) of uncertain significance.

The genetic testing algorithm is dependent upon the reason for referral, sample type received and laboratory service arrangements. The following paragraphs outline strategies for diagnostic testing in a proband, family testing when the familial causative genetic variant(s) is known, and testing in the context of reproductive options for couples with a family history of mitochondrial disease.

### Molecular testing in a proband with suspected mitochondrial disease

There are two main alternative approaches/strategies for genetic testing of patients with suspected mitochondrial disease: (1) targeted testing of ‘common’ mtDNA variants and/or targeted nuclear testing, followed by more comprehensive testing if required and if resources allow; (2) NGS of the mitochondrial genome and/or nuclear genes, e.g., by whole exome sequencing (WES) or whole genome sequencing (WGS).

First-line targeted testing of blood DNA can be appropriate for routine referrals (where there is not an urgent clinical need to obtain a diagnosis), for clinical presentations which are highly suggestive of a particular variant or gene (e.g., MELAS, MERRF, LHON, Pearson syndrome, *POLG*-related disorders), and/or where resources are limited. In patients where no pathogenic variant is detected in blood, additional clinical information and samples (e.g., urine) or a muscle biopsy for biochemical and histopathological investigations may be suggested. Further genetic testing may be carried out in a stepwise approach depending on the remaining suspicion of mitochondrial disease.

Alternatively, comprehensive NGS-based testing can be adopted for sensitive and efficient first-line genetic testing. This approach can be followed for all referral indications but is particularly appropriate for more complex phenotypes and/or for urgent referrals. As both mtDNA disease and nuclear mitochondrial disease account for a significant proportion of childhood-onset and adult-onset mitochondrial disorders [1, 2], simultaneous testing of both mtDNA and nuclear DNA is recommended if possible. However, if a sequential approach is required to reduce costs, then it is generally most appropriate to undertake mtDNA testing first due to the lower cost and shorter turnaround time of whole mitochondrial genome NGS compared to large nuclear gene panel sequencing (by panel-based NGS, WES or WGS). For urgent paediatric referrals where samples from a parent–child trio can be obtained, gene-agnostic WES or WGS, where data is analysed with no gene panels or other preconceptions about where variants associated with a condition are located, is an appropriate testing strategy. While WGS approaches typically include deep sequencing of the whole mitochondrial genome (with appropriate bioinformatic analysis), a gene-agnostic trio WES may require additional whole mtDNA NGS to reliably screen the mitochondrial genome with sufficient sensitivity, although studies have shown that off-target reads from exome sequencing may be adequate [21] (discussed further in the ‘Genetic testing methodologies and approaches’ section). Gene-agnostic trio WGS/WES may also be appropriate for referrals where mitochondrial disease is one of several possible differential diagnoses.

**Fig. 1:**
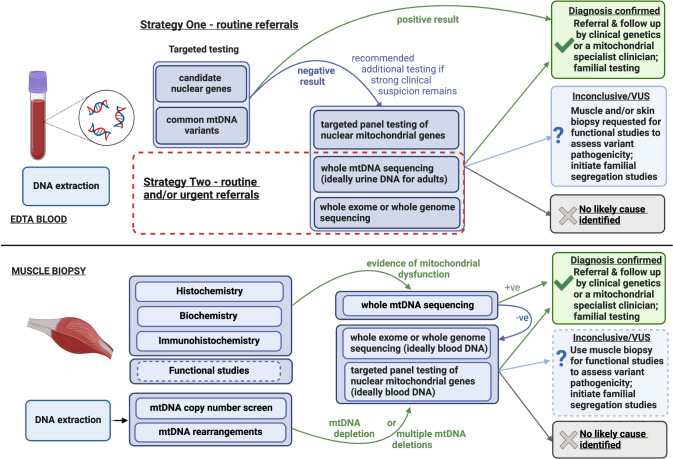
Overview of genetic testing strategies for mitochondrial disease patients. Upper panel: blood sample received. Strategy 1, a more targeted approach, may be appropriate for routine referrals or where there are barriers to parallel testing. Strategy 2 permits a more rapid and thorough, but more expensive, testing strategy and is amenable to both routine and clinically urgent cases, such as critically ill paediatric patients. When adopting Strategy 2, simultaneous testing of both mtDNA and nuclear DNA is recommended if resources allow; if sequential testing is required, mtDNA testing can be prioritised due to reduced cost and turnaround time. Lower panel: muscle biopsy received. As muscle biopsy is no longer widely used in first-line diagnostics, this pathway will typically apply if genetic analysis of blood DNA does not identify a cause or is inconclusive.

A flowchart of the recommended molecular genetic testing algorithms in referrals from patients with suspected mitochondrial disease is shown (Fig. 1) with further detail provided below. Although there will be some differences in genetic testing pathways between centres worldwide, the UK algorithms presented here are broadly applicable and are consistent with international pathways published for adult-onset mitochondrial disease [22].

#### Mitochondrial DNA testing in a proband with suspected mitochondrial disease

Pathogenic mtDNA variants can be SNVs or small indels affecting either protein-coding or mitochondrial tRNA variants, or large-scale mtDNA rearrangements such as the ‘common’ 4977 bp mtDNA deletion, though these may be highly variable in size [23, 24].

As a consequence of the transition to a ‘genomics first’ diagnostic approach, mtDNA testing is typically undertaken early in the diagnostic pathway via analysis of blood and/or urine DNA, as outlined in the algorithm (Fig. 1). However, it is important to note that muscle remains the preferred sample type for detection of some mtDNA variants (e.g., *MT-TP* m.15975T>C) and for large-scale mtDNA rearrangements; therefore, muscle biopsy may be required if blood/urine analysis does not identify a genetic diagnosis.

MtDNA testing is increasingly moving towards first-line whole mtDNA sequencing by mtDNA NGS, WES or WGS, as NGS costs continue to drop and bioinformatic pipelines become increasingly efficient and sophisticated. WES and WGS capture allow simultaneous analyses of both nuclear and mtDNA genes, as discussed above. Nevertheless, targeted testing for common mtDNA variants and large-scale mtDNA rearrangements can still be appropriate and cost-effective. Table 1 provides recommendations for the minimum level of appropriate targeted mtDNA testing for the most common diagnostic referral reasons, as well as guidance on more extensive testing that may be warranted.

**Table 1:** Mitochondrial DNA testing for common diagnostic referral reasons.

Phenotype/syndrome	Phenotype details	Minimum level of testing	Tissue type^a^	Possible further testing
Ataxia	Possible diagnosis of: late-childhood or adult-onset peripheral neuropathy, ataxia, pigmentary retinopathy (NARP) OR Mitochondrial myopathy, encephalopathy, lactic acidosis, and stroke-like episodes (MELAS). Ataxia may be present in up to half of MELAS patients.	m.8993T>G p.(Leu156Arg) m.8993T>C p.(Leu156Pro) (*MT-ATP6*) + m.3243A>G (*MT-TL1*)	Any (e.g., blood) Muscle > Urine > Blood (a normal result in blood from individuals aged ≥18 years does not exclude presence in another tissue)	Nuclear DNA testing may also be appropriate Large-scale mtDNA rearrangement testing Whole mtDNA sequencing (e.g., after further clinical examination and/or muscle biopsy)
Cardiomyopathy	Familial hypertrophic cardiomyopathy with maternal inheritance	m.4300A>G (*MT-TI*) + m.3243A>G (*MT-TL1*)	Any (e.g., blood) Muscle > Urine > Blood (a normal result in blood from individuals aged ≥18 years does not exclude presence in another tissue)	Nuclear DNA testing may also be appropriate Whole mtDNA sequencing (e.g., after further clinical examination and/or muscle biopsy)
Diabetes and hearing loss	Diabetes mellitus and sensorineural hearing loss	m.3243A>G (*MT-TL1*)	Urine > Blood (a normal result in blood from individuals aged ≥18 years does not exclude presence in another tissue)	Large-scale mtDNA rearrangement testing Nuclear DNA testing may also be appropriate Whole mtDNA sequencing (e.g., after further clinical examination)
Encephalopathy/seizures with lactic acidosis	Possible diagnosis of: mitochondrial myopathy, encephalopathy, lactic acidosis, and stroke-like episodes (MELAS) OR Infantile onset subacute relapsing encephalopathy, cerebellar and brain stem signs (MILS)	m.3243A>G (*MT-TL1*) + m.8993T>G p.(Leu156Arg) m.8993T>C p.(Leu156Pro) (*MT-ATP6*)	Muscle > Urine > Blood (a normal result in blood from individuals aged ≥18 years does not exclude presence in another tissue) Any (e.g., blood)	Nuclear DNA testing may also be appropriate Large-scale mtDNA rearrangement testing Whole mtDNA sequencing (e.g., after further clinical examination and/or muscle biopsy)
Hearing loss	Non-syndromic sensorineural hearing loss, particularly if onset following aminoglycoside exposure	m.1555A>G (*MT-RNR1*)	Any (e.g., blood)	Nuclear DNA testing may also be appropriate Whole mtDNA sequencing may be appropriate if there is a maternal family history
Kearns–Sayre syndrome	Onset below the age of 20 years: PEO and pigmentary retinopathy with one of either cardiac conduction block, cerebrospinal fluid protein concentration greater than 100 mg/dL, or cerebellar ataxia	Large-scale mtDNA rearrangements (single and multiple deletions) + m.3243A>G (*MT-TL1*)	Muscle > Urine > Blood (may be detectable in blood but a normal result does not exclude presence in another tissue) Muscle > Urine > Blood (a normal result in blood from individuals aged ≥18 years does not exclude presence in another tissue)	Nuclear DNA testing may also be appropriate Whole mtDNA sequencing (e.g., after further clinical examination and/or muscle biopsy)
Leber hereditary optic neuropathy, LHON, optic atrophy	Childhood or midlife (adult-onset) acute or subacute painless bilateral central vision loss	m.3460G>A p.(Ala52Thr) (*MT-ND1*) m.11778G>A p.(Arg340His) (*MT-ND4*) m.14484T>C p.(Met64Val) (*MT-ND6*)	Blood	Nuclear optic atrophy genes Whole mtDNA sequencing (e.g., after further clinical examination)
Myoclonic epilepsy	Myoclonus, seizures; Cerebellar ataxia; Myopathy	m.8344A>G (*MT-TK*) + m.3243A>G (*MT-TL1*)	Any (e.g., blood) Muscle > Urine > Blood (a normal result in blood from individuals aged ≥18 years does not exclude presence in another tissue)	Nuclear DNA testing may also be appropriate Whole mtDNA sequencing (e.g., after further clinical examination and/or muscle biopsy)
Pearson syndrome	Sideroblastic anaemia of childhood; Pancytopenia; Exocrine pancreatic failure	Large-scale mtDNA rearrangements	Any (e.g., blood)	N/A
Progressive external ophthalmoplegia (PEO), ptosis	Typically, adult-onset ptosis, paralysis of the extraocular muscles (ophthalmoplegia), oropharyngeal weakness, and variably severe proximal limb weakness	Large-scale mtDNA rearrangements (single and multiple deletions) + m.3243A>G (*MT-TL1*)	Muscle > Urine > Blood (blood is not suitable from patients aged >20 years) Muscle > Urine > Blood (a normal result in blood from individuals aged ≥18 years does not exclude presence in another tissue)	Nuclear DNA testing may also be appropriate Whole mtDNA sequencing (e.g., after further clinical examination and/or muscle biopsy)
Stroke-like episodes	Stroke-like episodes, typically before age 40 years	m.3243A>G (*MT-TL1*)	Muscle > Urine > Blood (a normal result in blood from individuals aged ≥18 years does not exclude presence in another tissue)	Nuclear DNA testing may also be appropriate Whole mtDNA sequencing (e.g., after further clinical examination and/or muscle biopsy)

Guidance on the minimum recommended level of mitochondrial DNA testing for the most common diagnostic referral reasons.

^a^‘>’ used to indicate the order of tissue type preference if all sample types are available; in reality, blood is typically tested first as the most readily available target DNA source.

#### Nuclear gene testing in a proband with suspected mitochondrial disease

Pathogenic variants in >300 nuclear-encoded genes are known to cause mitochondrial disease and may be associated with autosomal dominant, autosomal recessive, X-linked or de novo inheritance patterns [25]. Nuclear genes encode proteins that are essential for mitochondrial structure and function. These include structural subunits, assembly factors and cofactors of the respiratory chain enzyme complexes, as well as proteins of the mtDNA translation machinery, mtDNA maintenance, mitochondrial import channels, and mitochondrial fission and fusion, among others [26]. Depending on the clinical indication, testing may be single gene based where a genotype-phenotype occurs, or more frequently as gene panel-based NGS, WES or WGS. When adopting gene panel analysis, it may be appropriate to analyse other gene panels for clinically similar but non-mitochondrial differential diagnoses (e.g., other metabolic disorders, leukodystrophies or cardiomyopathies); in some cohorts, non-mitochondrial diagnoses may even outnumber mitochondrial diagnoses [27]. Gene-agnostic WGS/WES is also increasingly being adopted, for which parent–child trio analysis is recommended where possible (as trio adds valuable data to aid variant interpretation). This could be either as a first-line test (for example, in urgent paediatric referrals), or an additional test for other referrals with a strong clinical suspicion of mitochondrial disease where gene panel-based testing has not identified a diagnosis.

Table 2 summarises nuclear-associated mitochondrial disorders and provides guidance on appropriate referrals for both single-gene testing (including common pathogenic variant screening within a single gene) and panel testing; the table also includes information on the minimal appropriate gene content of panels.

**Table 2:** Nuclear gene testing in a proband with suspected mitochondrial disease.

Clinical indication/disorder	Phenotype/*appropriate referral reasons*	Gene(s)—for gene panels, genes based on PanelApp gene content are listed^a^	Function	Possible further testing, especially mtDNA
*Single gene tests*				
*POLG*-related disorders (AR, rarely AD) *Common variant testing may be prioritised (refer to the main text for further details)*	Highly variable from Alpers syndrome to PEO *Epilepsy, neuropathy, ataxia, suspected mtDNA maintenance disorder, sodium valproate toxicity*	*POLG*	Catalytic subunit of DNA polymerase gamma, required for replication of mtDNA	Nuclear gene panels as appropriate Refer to phenotypes in Table 1 for appropriate mtDNA testing
Mitochondrial neurogastrointestinal encephalomyopathy (MNGIE) (AR)	Progressive gastrointestinal dysmotility, PEO, leukoencephalopathy, demyelinating peripheral neuropathy, distal weakness *Elevated plasma levels of thymidine/deoxyuridine* *Reduced TP activity*	*TYMP*	Thymidine phosphorylase (TP), catalyses phosphorylation of thymidine/deoxyuridine to thymine/uracil	m.3243A>G (*MT-TL1*) *POLG* Mitochondrial DNA maintenance disorder gene panel
Thiamine metabolism dysfunction syndrome-2 (also known as biotin-responsive basal ganglia disease) (AR)	Childhood-onset encephalopathy *Strong clinical suspicion*	*SLC19A3*	Thiamine transporter	Nuclear gene panels as appropriate Refer to phenotypes in Table 1 for appropriate mtDNA testing
Mitochondrial complex V deficiency nuclear type 2 (AR) *Common variant testing may be prioritised (refer to the main text for further details)*	Neonatal mitochondrial encephalo-cardiomyopathy *Strong clinical suspicion, particularly of Roma population origin* *Complex V deficiency*	*TMEM70*	Mitochondrial membrane protein involved in the biogenesis of mitochondrial ATP synthase	Nuclear gene panels as appropriate Refer to phenotypes in Table 1 for appropriate mtDNA testing
*Nuclear gene panels*				
Mitochondrial liver disease, including transient infantile liver failure (AR)	Liver disease *Infantile acute liver failure* *Transient infantile liver failure* *Liver disease with suspected mitochondrial dysfunction*	*BCS1L*	OXPHOS assembly factor	mtDNA replication and maintenance
		*DGUOK, MPV17, POLG, TWNK*	mtDNA replication and maintenance	
		*TRMU*	tRNA modification	
Mitochondrial DNA maintenance disorder (AR, AD)	MtDNA depletion syndromes, PEO with multiple mtDNA deletions *Evidence of mtDNA depletion or multiple mtDNA deletions* *Strong clinical suspicion*	*AFG3L2, SPG7*	Mitochondrial protein quality control	mtDNA copy number analysis Large-scale mtDNA rearrangements
		*ABAT, DGUOK, DNA2, MGME1, MPV17, POLG, POLG2, RNASEH1, RRM2B, SLC25A4, SUCLA2, SUCLG1, TK2, TOP3A, TWNK, TYMP*	mtDNA replication and maintenance	
		*MFN2, OPA1*	Mitochondrial dynamics	
		*DNM2, FBXL4*	Other	
Mitochondrial disorder with complex I deficiency (AR, rarely X-linked)	Mainly neonatal/childhood-onset Leigh syndrome, neurological disorder or cardiomyopathy *Biochemical evidence of complex I deficiency*	*NDUFA1, NDUFA10, NDUFA11, NDUFA2, NDUFA6, NDUFA9, NDUFB11, NDUFB3, NDUFB8, NDUFS1, NDUFS2, NDUFS3, NDUFS4, NDUFS6, NDUFS7, NDUFS8, NDUFV1, NDUFV2*	OXPHOS Complex I subunits and accessory subunits	Whole mtDNA sequencing Comprehensive mitochondrial disorder nuclear gene panel
		*ACAD9, FOXRED1, NDUFAF1, NDUFAF2, NDUFAF3, NDUFAF4, NDUFAF5, NDUFAF6, NDUFAF8, NUBPL, TMEM126B*	OXPHOS Complex I assembly factors	
Mitochondrial disorder with complex II deficiency (AR, rarely AD)	Leukoencephalopathy, Leigh syndrome^b^ *Biochemical evidence of complex II deficiency*	*SDHA, SDHD*	OXPHOS Complex II subunits	Comprehensive mitochondrial disorder nuclear gene panel
		*SDHAF1*	OXPHOS Complex II assembly factors	
Mitochondrial disorder with complex III deficiency (AR)	Variable including Leigh syndrome, neurological disorder, liver disease, renal tubular acidosis *Biochemical evidence of complex III deficiency*	*CYC1, UQCRB*	OXPHOS Complex III subunits	Whole mtDNA sequencing Comprehensive mitochondrial disorder nuclear gene panel
		*BCS1L, LYRM7, TTC19, UQCC2*	OXPHOS Complex III assembly factors and chaperones	
Mitochondrial disorder with complex IV deficiency (AR)	Mainly Leigh syndrome, cardiomyopathy. *Biochemical evidence of complex IV deficiency*	*COX6A1, COX6B1, COX7B*	OXPHOS Complex IV subunits	Whole mtDNA sequencing Comprehensive mitochondrial disorder nuclear gene panel
		*COA6, COA7, COX10, COX14, COX15, COX20, NDUFA4, PET100, SURF1*	OXPHOS Complex IV assembly factors and chaperones	
		*FASTKD2, LRPPRC*	RNA processing/modification and transcriptional regulation	
		*APOPT1, SCO1, SCO2, TACO1*	Other	
Mitochondrial disorder with complex V deficiency (AR)	Mainly Leigh syndrome, cardiomyopathy *Biochemical evidence of complex V deficiency*	*ATP5F1D*	OXPHOS Complex V subunits	Whole mtDNA sequencing Comprehensive mitochondrial disorder nuclear gene panel
		*ATPAF2, TMEM70*	OXPHOS Complex V assembly factors and chaperones	
Pyruvate dehydrogenase (PDH) deficiency (X-linked, AR, rarely AD)	Variable including Leigh syndrome, neurological disorder, dysgenesis of the corpus callosum *Strong clinical suspicion* *Biochemical evidence of PDH deficiency*	*DLAT, DLD, PDHA1, PDHB, PDHX*	Core subunits of the PDH complex	Comprehensive mitochondrial disorder nuclear gene panel
		*PDP1*	PDH regulation	
		*BOLA3, GLRX5, IBA57, ISCA1, ISCA2, NFU1*	Iron-sulfur (Fe-S) biosynthesis^d^	
		*LIAS, LIPT1, LIPT2, TPK1*	Cofactor biosynthesis^d^	
		*SLC19A2, SLC19A3, SLC25A19, SLC25A26*	Transporters required for cofactor metabolism^d^	
		*ECHS1, FBXL4, HIBCH, LONP1*	Other/unknown^d^	
Comprehensive mitochondrial disorder nuclear gene panel^c^ (AR, AD, X-linked)	Highly variable *Strong clinical suspicion of mitochondrial disease (where a nuclear defect is suspected and/or mtDNA variants have been excluded)* *Biochemical evidence of combined respiratory chain enzyme deficiency*	All genes listed in panels above		
		*AIFM1, CLPB, CLPP, DNAJC19, GFER, HSPD1, HTRA2, MIPEP, PMPCA, PMPCB, SACS*	Protein processing	
		*ELAC2, GTPBP3, HSD17B10, MTFMT, MTO1, MTPAP, PNPT1, PUS1, TRIT1, TRMT10C, TRMT5, TRNT1*	RNA processing/modification	
		*AARS2, CARS2, DARS2, EARS2, FARS2, GARS1, HARS2, IARS2, KARS1, LARS2, MARS2, NARS2, PARS2, QRSL1, RARS2, SARS2, VARS2, WARS2, YARS2*	Mitochondrial aminoacyl tRNA synthetases	
		*MRPS2, MRPS22, MRPS34, MRPL3, MRPL44*	Mitochondrial ribosome components	
		*MTRFR, GFM1, GFM2, RMND1, TSFM, TUFM*	Translation	
		*AGK, ATAD3A, C19orf70, DNM1L, GDAP1, MFF, MSTO1, OPA3, PNPLA8, SERAC1, SLC25A46, TAZ*	Mitochondrial membrane and dynamics	
		*FDX2, FDXR, FLAD1, HCCS, HLCS, ISCU, MECR, NADK2, NAXE, PDSS1, PDSS2*	Cofactors	
		*ABCB7, SLC25A1, SLC25A12, SLC25A3, SLC25A32, SLC25A38, SLC25A42, TIMM50, TIMM8A*	Carriers, transporters, protein import	
		*COQ2, COQ4, COQ6, COQ7, COQ8A, COQ8B, COQ9*	Coenzyme Q10 biosynthesis	
		*ACO2, CA5A, FH, MDH2, MPC1, PC, PPA2*	PDC and TCA cycle	
		*ETFDH*	Fatty acid β-oxidation	
		*BTD*	Biotin synthesis	
		*ANO10, APTX, C1QBP, CHCHD10, ETHE1, MICU1, RTN4IP1, SFXN4*	Other	

Single nuclear gene and panel tests for routine diagnostic referrals of patients with suspected primary mitochondrial disease, including guidance on appropriate referrals and information on minimal appropriate gene content of panels.

*AR* autosomal recessive, *AD* autosomal dominant, *PEO* progressive external ophthalmoplegia.

^a^PanelApp: https://nhsgms-panelapp.genomicsengland.co.uk/ and https://panelapp.genomicsengland.co.uk/panels/. PanelApp ‘green’ genes are listed, from the NHS Genomic Medicine Service Signed Off Panels Resource as of 5 November 2022 (these are versions 1.2 for all panels except version 1.17 for the Comprehensive mitochondrial disorder nuclear gene panel, which is termed ‘possible mitochondrial disorder—nuclear genes’ on PanelApp). Laboratories may wish to include additional genes in these panels, such ‘amber’ genes with moderate/limited evidence of gene-disease association and candidate genes based on known biological function but with no reported disease association to date. Content of nuclear gene panels requires regular updates as new evidence of gene-disease associations is identified. Users can refer to the PanelApp website for updates and for gene content of the latest signed-off versions for UK diagnostic use.

^b^Mono-allelic dominant SDHx variants are associated with predisposition to cancer (pheochromocytoma and paraganglioma) and are reviewed in [90]. Complex II associated genes that are currently listed in OMIM with mitochondrial-related disease association include *SDHA*, *SDHD* and *SDHAF1*.

^c^Gene-agnostic WES or WGS is an appropriate alternative testing strategy, if available, particularly for urgent paediatric referrals where samples from a parent–child trio can be obtained and/or for referrals where mitochondrial disease is one of several possible differential diagnoses.

^d^These genes also function in pathways unrelated to pyruvate dehydrogenase.

Specific single gene testing remains a valuable simple and rapid first-line test when the associated disorder is common, for treatable disorders with a characteristic phenotype, when there are common founder variants(s), and/or if biochemical evidence points to a defect in the particular gene.

- *POLG*-related disorders represent one of the most common inherited mitochondrial disorders. Screening of four autosomal recessive pathogenic variants/alleles in the *POLG* gene that are common in the European population may be prioritised in the first instance: c.1399G>A p.(Ala467Thr), c.1760C>T p.(Pro587Leu) in *cis* with c.752C>T p.(Thr251Ile), c.2243G>C p.(Trp748Ser), and c.2542G>A p.(Gly848Ser) (NM_002693.3) [28–30].
- Biallelic pathogenic variants in *TYMP*, which encodes thymidine phosphorylase (TP), cause mitochondrial neurogastrointestinal encephalomyopathy (MNGIE). Biochemical results, i.e., elevated plasma levels of thymidine and deoxyuridine and/or reduced TP enzyme activity, may point to a defect in *TYMP*.
- Biallelic pathogenic variants in *SLC19A3* cause thiamine metabolism dysfunction syndrome-2 (THMD2), also known as biotin-responsive basal ganglia disease (BBGD). Early administration of high doses of biotin can result in clinical improvement.
- Autosomal recessive *TMEM70*-related complex V deficiency, also known as mitochondrial complex V (ATP synthase) deficiency nuclear type 2 (MC5DN2), is common in the Roma population. A founder pathogenic variant in this population has been described and may be screened initially if appropriate (c.317-2A>G NM_017866.6) [31].
  Nuclear gene analysis may be more comprehensively performed by interrogation of multi-gene panels by next-generation sequencing. The genes included in these panels are collated based on related clinical phenotypes. Within the UK, PanelApp [32] offers a valuable resource for the curation of nuclear gene panels for genetic testing, including mitochondrial disorders. These panels are regularly reviewed and updated in light of current literature and data from diagnostic and research laboratories. As indicated above, due to clinical overlap with non-mitochondrial disorders, it may be appropriate to simultaneously analyse other gene panels, particularly if WES or WGS is undertaken.

For nuclear mitochondrial disorders, nuclear gene panels can be subdivided as follows:

- Comprehensive mitochondrial disorder nuclear gene panel, including all known nuclear genes associated with Mendelian mitochondrial disease; with the shift in paradigm from ‘biopsy first’ to ‘genomics first’, this panel is now becoming the most relevant test for patients with a strong clinical suspicion of mitochondrial disease (where a nuclear defect is suspected).
- Targeted nuclear gene panels when there is a specific enzyme deficiency, evidence of an mtDNA maintenance defect, or specific phenotype:
  - OXPHOS complexes, assembly factors and ancillary proteins, i.e., gene panels for complexes I, II, III, IV and V deficiency.
  - mtDNA maintenance disorders, due to a diverse group of nuclear genes that function in several aspects of maintenance of mtDNA including replication, maintenance of nucleotide pools, and mitochondrial fusion and fission; pathogenic variants in these genes result in defective mtDNA synthesis, causing accumulation of multiple deletions and/or depletion and ultimately impaired OXPHOS energy production.
  - Liver presentations, particularly appropriate in infants with acute liver failure where mitochondrial liver disease is suspected/possible.
  - Pyruvate dehydrogenase (PDH) deficiency, due to defects in genes encoding the core subunits of PDH or proteins involved in the regulation or biosynthesis of cofactors.

### Family testing

Following a genetic diagnosis of mitochondrial disease, it is recommended that the proband (and their family) are referred to clinical genetics or a specialist mitochondrial service for appropriate genetic counselling and family follow-up. Recurrence risk and risk to other family members is dependent on whether the diagnosis is due to a nuclear-encoded gene or an mtDNA variant.

In families with a nuclear gene diagnosis of mitochondrial disease, counselling and further family testing is related to the mode of inheritance of the specific gene (autosomal dominant, autosomal recessive or X-linked).

In families with an mtDNA-related diagnosis, inheritance may be de novo or matrilineal and male patients are not at risk of transmitting mtDNA variants to their offspring.

Testing of asymptomatic children below the age of 16 that have a family history of mtDNA-related mitochondrial disease is particularly challenging and routine practice in the UK is not to test these children. Clinical investigations such as an echocardiogram may be performed in children over the age of 5 years, to ensure there is no evidence of cardiomyopathy. Continued cardiac monitoring may be offered and this should be considered on a case-by-case basis. It is recommended that laboratories discuss referrals for genetic testing of asymptomatic children with specialised clinicians or clinical geneticists wherever possible prior to testing.

Additional considerations of the familial risk of mtDNA variants are detailed below.

#### Mitochondrial DNA single-nucleotide variants (SNVs) and duplications

These may be transmitted through the maternal line. The risk of inheritance to other family members depends on the genetic status of the proband’s mother. If she is heteroplasmic for a pathogenic mtDNA variant all her maternal relatives may be at risk of also having the variant. The variant level in the mother of the proband, influences her risk of developing symptoms and the risk of transmission to any other offspring she may have, though this is unpredictable due to the mitochondrial bottleneck. The clinical variability that may be observed within the same family may be due to variation in mutation load and tissue distribution differences between sibs and other maternal family members [33]. For females who are homoplasmic for a pathogenic mtDNA variant, all their offspring will be expected to inherit the variant. Variable penetrance and clinical variability can also be observed for homoplasmic variants (e.g., LHON-associated pathogenic variants and also *MT-TI* m.4300A>G).

#### Single mtDNA deletions

These are generally sporadic (de novo) and therefore in the absence of any family history the risk of recurrence to any siblings of the proband is considered low. A clinically affected female with an mtDNA deletion is estimated to have a 1 in 24 risk of transmitting the deletion to her offspring [34].

#### Testing of extended family members

Maternal relatives may be at risk of inheriting the familial mtDNA pathogenic variant and therefore testing can be offered as appropriate. Ideally, cascade testing of closely related family members should be carried out first in order to inform risks to more distant relatives.

### Reproductive options and associated genetic testing

#### Reproductive options and prenatal diagnosis for nuclear variants

Reproductive options and genetic counselling for couples with a known family history of mitochondrial disease due to a nuclear gene defect is similar to other Mendelian conditions. For autosomal recessive conditions, it is recommended to confirm parental carrier status prior to offering prenatal diagnosis (PND). PND may be offered by chorionic villous sampling (CVS, usually between 11 and 12 weeks gestation) or by amniocentesis usually later in pregnancy (15–17 weeks gestation). Testing of the CVS or amniocentesis may be carried out on direct extractions or cultured cells either by Sanger sequencing or other targeted approaches. It is essential that testing for maternal cell contamination (MCC) is also carried out on the foetal DNA tested to exclude gross contamination that may affect the interpretation of the results. Guidelines for such testing are presented elsewhere [35]. Preimplantation genetic diagnosis may be also available in specialised laboratories usually following national guidelines for authorisation and approval of such tests.

#### Reproductive options and prenatal diagnosis for mtDNA variants

Due to the strict maternal inheritance of mtDNA, male patients harbouring a pathogenic mtDNA variant can be reassured that they are not at risk of transmitting the variant to their offspring. Genetic counselling in female patients is often challenging due to the complexities of mtDNA variant transmission resulting from the effect of mtDNA bottleneck during oogenesis and prediction of disease risk. Available reproductive options may also differ depending on the pathogenic variant involved and the variant level. It is recommended that advice is offered by clinical genetics clinics or specialised mitochondrial services where all available reproductive options can be evaluated. In addition, it is recommended that wherever possible, such counselling starts early in a woman’s reproductive life as options can become limited with advanced age and decline in ovarian reserve [36]. The European Neuro Muscular Centre (ENMC) international workshops have been valuable in developing guidelines for the management of reproductive options in mitochondrial disease which may be of interest to those wishing to explore this further [37].

The evaluation of risk of disease transmission in women harbouring heteroplasmic pathogenic mtDNA variants when seeking prenatal diagnosis, should include assessment of variant levels in at least two different tissues (e.g., blood, urine, buccal) and where possible, by testing other maternal relatives and any affected offspring. This testing should be offered ideally in advance.

De novo occurrences of pathogenic mtDNA variants have been reported [38]; however, it is important to consider that the variant may be below the level of detection in the maternal tissues tested and the possibility of maternal germline mosaicism cannot be excluded.

#### Prenatal diagnosis by CVS or amniocentesis

Prenatal testing for the prevention of mitochondrial DNA disease is a reproductive option that is available to some females at risk of transmitting an mtDNA pathogenic variant to their offspring. For a number of common mtDNA variants studied, it has been shown that the variant levels remain stable in the developing foetus after approximately 10 weeks of gestation [39–41]; therefore, the prenatal sample allows the risk of mitochondrial disease in the resulting child to be estimated.

It is vital that prenatal testing for mtDNA variants should be offered only on direct CVS/amniocytes and not on cultured cells [33, 42] as levels of mtDNA variants can change during culture. Recent publications [41, 43] have reported a small number of cases with high variance between CVS heteroplasmy and postnatal test results and also within placental samples from the same pregnancy; these results can be considered in the context of offering CVS, however, in our long-standing experience (unpublished findings), we have not encountered any significant discrepancies between levels of CVS heteroplasmy and prenatal outcome.

##### Heteroplasmic mtDNA variants

Prenatal diagnosis can be informative for certain heteroplasmic mtDNA variants [44]. Prediction of risk from mutant load can be difficult, in particular, where the correlation between variant levels and disease severity is complex. For the *MT-TL1* m.3243A>G pathogenic variant, a model has been developed to enable predictions of likely outcomes below a specified threshold based on maternal m.3243A>G level [45]. For other variants with distinct segregation patterns (e.g., *MT-ATP6* m.8993T>G p.(Leu156Arg)) a reliable prediction may be possible for most mutant loads [46]. For variants present at a high level in maternal DNA, prenatal diagnosis is generally not appropriate. However, a few exceptions have been described where markedly skewed variant levels have been observed in the oocytes from a female with high mutant loads [47], and prenatal diagnosis may be informative. For apparently de novo mtDNA variants, prenatal diagnosis may be available if requested for reassurance.

In addition, females with a family history of a pathogenic mtDNA variant and undetectable mutant load in blood, have been reported with a low risk of having an affected pregnancy [41]. Prenatal diagnosis is still available for these females for reassurance, as the familial mtDNA variant may be below the level of detection in blood or may be restricted in the germline.

##### Homoplasmic mtDNA variants

Prenatal diagnosis is generally not appropriate for any homoplasmic variants (see the section ‘Mitochondrial donation (mitochondrial replacement therapy [MRT])’). Prenatal diagnosis for the *MT-RNR1* m.1555A>G variant, which is associated with susceptibility to aminoglycoside-induced ototoxicity and non-syndromic sensorineural deafness, is not considered to be appropriate, as this homoplasmic variant is not associated with a severe neurological phenotype and testing can be performed postnatally, potentially as a point of care test [48], to inform tailored antibiotic prescription.

##### Single, large-scale mtDNA deletions

Single, large-scale mtDNA deletions are often sporadic and thus the recurrence risk is low. Risk estimates were evaluated in a study of 226 families where a single mtDNA deletion had been detected in the proband [34]. Clinically unaffected women were highly unlikely to have more than one affected child and clinically affected women had a 1 in 24 risk of having a clinically affected child. Prenatal diagnosis may be available if requested for reassurance.

#### Preimplantation genetic diagnosis (PGD)

PGD for mtDNA variants involves quantification of the level of a pathogenic mtDNA variant to allow for the selection of the most appropriate embryo(s), based on mutant load and quality, for transfer. PGD is mainly performed on a single blastomere from cleavage-stage embryos, with the mutant load level in the biopsied cell indicative of the mutant load level within the embryo [49]. Limited data exist for an alternative approach at the blastocyst stage, with one report presenting inconsistent results between the biopsy and postnatal outcomes [50]. Foetal variant levels would be required to be below the critical threshold for severe disease expression [25] and the prediction of the percentage threshold below which clinical symptoms are unlikely to occur later in adult life is challenging. A threshold of ≤18% has been proposed [51]. However, the exact threshold would need to be considered on a case-by-case basis as it can be variable depending on the familial mtDNA variant involved, previous familial transmission, clinical presentations between family members, and local policies and procedures of the centre offering the PGD service. Therefore, provision of PGD should only be offered within specialised centres where comprehensive investigations of the threshold in each family can be undertaken. Moreover, as PGD is a risk reduction procedure it may only benefit women who are likely to produce oocytes with low mutant load, further highlighting that specialist reproductive counselling is required. PGD is not indicated for women that harbour variants at high mutant load (with the exception of cases of variants associated with distinct segregation patterns such as *MT-ATP6* m.8993T>G/C or m.9176T>C/G variants [47, 52]).

#### Mitochondrial donation (mitochondrial replacement therapy [MRT])

Mitochondrial donation is an in vitro fertilisation-based technique that can be offered to women harbouring pathogenic mtDNA variants where other options are not viable (e.g., for homoplasmic variants and/or heteroplasmic variants at high mutant load) and in countries/jurisdictions (such as the UK) where the technique has been legalised. The two techniques currently used, maternal spindle transfer (MST) and pronuclear transfer (PNT) are reviewed in [25]. The use of this intervention is subject to national legislation and guidelines. Further information regarding the availability and suitability of this procedure should be sought from the patient’s local clinical genetics services.

## Genetic testing methodologies and approaches

### Testing of mitochondrial DNA

#### Sample type considerations

The likelihood of detecting a variant in certain referral categories is dependent on the sample type and/or the age of the patient. Therefore, both need to be taken into account when advising on the most appropriate sample type. Details of preferred sample types for different variants are given in Table 1, although blood is typically tested first as the most readily available target DNA source and a diagnosis can often be made preventing the need for invasive samples such as muscle biopsy.

In general, it is important to consider the following:

- Levels of some variants, such as *MT-TL1* m.3243A>G, decline in blood with increasing age; the proportion of m.3243A>G in blood DNA is reported to decline at a rate of approximately 1% per year [12] and so may not be detected in older patients with milder presentations. In our experience, patients aged over 40 years with mild symptoms, such as diabetes and deafness, often have 1–5% heteroplasmy in blood DNA, which may be missed depending on the testing methodology used.
- mtDNA rearrangements are also selected against and lost in rapidly dividing cells such as lymphocytes, thus unlikely to be detected in blood from adults that are >20 years of age [53]. NGS approaches raise the possibility of increased sensitivity to detect mtDNA rearrangements in blood; however, it is currently unclear whether this will enable reliable detection of mtDNA deletions/rearrangements in blood from adults and further studies are required.
- Urine can be a more appropriate source tissue compared to blood and is less invasive than muscle especially for *MT-TL1* m.3243A>G testing, as levels in urine correlate more closely with levels in muscle and are stable over time [54]. Referral of 20 ml of an early morning sample would be sufficient for most applications [55], although in our experience DNA quality and quantity is highly variable and the failure rate in downstream analyses is higher than for blood or muscle. In addition, detection of single deletions may be possible in urine [56, 57]. However, it should be noted that in cases with a negative result and where a strong clinical suspicion remains, the referral of a muscle biopsy should be recommended.
- Muscle is a post-mitotic and often clinically affected tissue and so is generally the optimal tissue for mitochondrial DNA analysis. In addition, muscle biopsy is extremely useful for histology, histochemistry and respiratory chain enzyme studies to help investigate a diagnosis of mitochondrial or other diseases. However, due to their invasive and costly nature, muscle biopsies are no longer routine first-line investigations. As discussed in the variant interpretation section below, muscle biopsy can be important for functional evaluation of variants of uncertain significance, both mtDNA and nuclear DNA (e.g., mtDNA maintenance disorders), and also to follow up (likely) pathogenic variants detected at low levels in other tissues where clinical significance to the patient’s symptoms may be uncertain. Muscle biopsies should be snap frozen in isopentane and delivered to testing laboratories on dry ice.
- Liver biopsies should also be snap frozen in isopentane and delivered to testing laboratories on dry ice. They provide an appropriate tissue for mtDNA depletion analysis in patients with liver disease. Similar considerations apply to liver biopsies as for muscle biopsies, although the biopsy procedure is more invasive and higher risk.

#### Testing for common mtDNA single-nucleotide variants

There are several methods that are currently available for testing for common mtDNA variants and the points given below should be considered when laboratories are considering assay selection [58]:

- Sensitivity and specificity: absence of false negatives and false positives respectively; ideally assays should have high sensitivity and specificity.
- Limit of detection: to test for heteroplasmic variants, ideally assays should be able to detect at least a level of 10%, although this may be variable depending on variant, sample type and reason for referral. For instance, a lower limit of detection would be required when testing for *MT-TL1* m.3243A>G in blood DNA from adults or familial testing for other heteroplasmic variants compared to the level required to screen for the common variants associated with LHON which are often homoplasmic.
- Quantitative: this is preferable for determining an accurate level of variant.
- Robustness: the selected method should have a low failure rate and be reproducible.

Different assays are available that meet some or all the criteria described above. These include:

- Pyrosequencing
- Real-time PCR
- Next-generation sequencing (NGS)
- Digital PCR/Droplet digital PCR
- Fluorescent restriction digest PCR followed by capillary electrophoresis
- Restriction digest PCR followed by agarose gel electrophoresis
- Sanger sequencing

Note that both restriction digest/agarose electrophoresis and Sanger sequencing methods are associated with a relatively high limit of detection and may risk the production of a false negative result. In addition, neither method allows variant levels to be quantified accurately.

Variants detected can be confirmed using an alternative method to exclude a false positive result. Common polymorphisms may interfere with analysis and lead to false positive or false negative results. For instance, the presence of the *MT-ATP6* m.8994G>A polymorphism may lead to a false negative result when testing for the *MT-ATP6* m.8993T>G/C variant by restriction digest PCR (RFLP) analysis [59]. Therefore, if this assay is used, testing for the polymorphism should also be undertaken, and if detected, then alternative analysis for *MT-ATP6* m.8993T>G/C should be performed. In addition, co-amplification of nuclear-encoded mitochondrial DNA segments (NUMTs) can lead to false positive results, particularly low-level heteroplasmy, if primers are not designed to avoid known NUMTs [60, 61].

#### Testing for mtDNA rearrangements

The key points listed above also apply in testing for mtDNA rearrangements. When testing muscle-derived DNA, the method employed should be able to detect both multiple mtDNA deletions and single mtDNA rearrangements. The limit of detection should be considered as single rearrangements in the blood may be present at a low level, even in children. Similarly, in patients with mitochondrial DNA maintenance disorders, levels of multiple mtDNA deletions may be low in muscle. Long-range PCR is a simple and quick method that is widely used for routine mtDNA rearrangement testing, however, heteroplasmy quantification and rearrangement breakpoint determination are not possible with this method. Determination of mtDNA rearrangement breakpoints [62] and the level of rearrangement [63] may be clinically useful for prognosis, or if the clinical phenotype is atypical, quantification may assist with interpretation.

Various methods are available which include:

- Long-range PCR: selection of primers is crucial and they should be able to detect the vast majority of mtDNA rearrangements (i.e., amplify most of the major arc [64]).
- Southern blotting: is useful for both quantification and characterisation of mtDNA rearrangements including deletions, duplications and deletion dimers [65].
- Real-time PCR/quantitative PCR: may be useful for quantification of mtDNA rearrangements [66].
- NGS/WGS: may be useful for determination of breakpoints and potentially quantification of mtDNA rearrangements (if a PCR-free library preparation method is used); also potential for increased sensitivity to detect low levels.

#### Testing for mtDNA depletion

MtDNA depletion analysis is a quantitative test to assess mtDNA copy number in a clinically affected post-mitotic tissue (such as muscle or liver) in patients with suspected mtDNA depletion syndrome (MDS). As a consequence of the increased availability of nuclear gene testing (to identify the primary genetic cause of mtDNA depletion syndromes) and reduced frequency of muscle biopsy early in the diagnostic pathway, mtDNA copy number analysis is now most often undertaken if a definitive diagnosis is not obtained from nuclear gene testing, i.e., if no genetic cause is identified or if VUS(s) is/are detected.

It is essential that data from normal control samples are used to determine the range of normal mtDNA copy number variation for each tissue type. In addition, an age-matched reference range may be necessary from normal controls, as there is evidence that mtDNA copy number changes with age [67, 68]. Currently, the most widely used methodology for routine referrals involves real-time PCR quantification relative to nuclear DNA. Other approaches include droplet digital PCR [69], Southern blotting, NGS/WGS.

Evidence of reduced mtDNA copy number in muscle or liver should be interpreted along with clinical, histological and respiratory chain enzyme findings and cannot unequivocally confirm a diagnosis of mtDNA depletion syndrome. In particular, this is due to the possibility of sampling issues and/or mtDNA depletion that is secondary to other non-mitochondrial disorders.

#### Whole mitochondrial genome sequencing analysis

Next-generation sequencing is preferred over Sanger sequencing as it more cost-effective due to its scalability and offers improved sensitivity in terms of the lower limit of detection (provided read depth is high) and accurate heteroplasmy quantification [70]. For instance, based on binomial distribution calculations at a read depth of 500, there is expected to be 99% probability that a 7.5% heteroplasmic variant will be detected in ≥5% of sequence reads. It is essential that laboratories also follow other relevant best practice guidelines that cover Sanger sequencing and NGS methodologies [71, 72]. Depending on local validation and reporting policies, reportable variants identified by NGS may need confirmation by an alternative method. With the continuous development of sequencing technologies, it is anticipated that alternative methodologies using emerging NGS/third-generation sequencing platforms will be utilised in the future for whole mitochondrial genome sequencing [73].

In addition to targeted NGS approaches, WES or WGS may be utilised to sequence the whole mitochondrial genome. Obtaining diagnostic-grade data (i.e., adequate sensitivity and accurate heteroplasmy quantification) from WES or WGS is dependent on read depth across the mitochondrial genome. MtDNA sequence data from WES arises from off-target reads [21] and is highly variable depending on the exome kit used. Therefore, adopting these approaches for diagnostic mitochondrial genome sequencing requires appropriate validation by the testing laboratory. Due to the possibility of false positive variant calls arising from NUMTs [74] confirmation of mtDNA variants by an alternative method is generally advisable.

### Testing of nuclear-encoded genes

There are no restrictions on the appropriate sample type. As the quantity of muscle and urine DNA is often limited and should be prioritised for mtDNA analysis, laboratories may need to request a blood sample for nuclear gene testing. As nuclear pathogenic variants follow all modes of inheritance, the techniques adopted for screening should be able to detect these with high sensitivity and specificity and any limitations noted. Screening for pathogenic variants in nuclear-encoded mitochondrial disease genes may be carried out by Sanger sequencing for single genes or by NGS for panels of genes (as detailed in the section ‘Reason for referral and genetic testing strategies—nuclear gene panels’). Generally, Sanger sequencing involves screening the genes coding exons plus intron/exon boundaries; however, for genes with known recurrent pathogenic variants outside these regions, screening should be extended (e.g., when there are known deep intronic variants [75]). For genes where recurrent pathogenic variants may be common in certain populations, targeted analysis (e.g., Sanger or pyrosequencing) can be carried out as a first-line test. This is best exemplified in *POLG* screening, where initial analysis for the four autosomal recessive pathogenic variants/alleles common in the European population may be prioritised prior to full gene sequencing (see the section ‘Nuclear gene testing in a proband with suspected mitochondrial disease’). NGS is generally used when the clinical indication requires several nuclear genes to be analysed simultaneously. Selection of relevant genes for panel design is detailed in the section ‘Reason for referral and genetic testing strategies—nuclear gene panels’. With NGS becoming more mainstream in diagnostic genetic laboratories in recent years, library preparation may utilise WES or WGS followed by targeted analysis of the genes of interest. This also offers the advantage of the application of other gene panels or periodic data re-analysis if a molecular diagnosis is not found. Alternatively, a targeted capture approach may be used. Best practice guidelines that cover Sanger sequencing and NGS methodologies validation are covered in [71, 72].

It is important to note that Sanger sequencing will not detect deletions or duplications involving one or more exons, though homozygous deletions could be suspected if they repeatedly fail to PCR amplify. Detection of these large-scale rearrangements requires copy number analysis, which is usually carried out by MLPA (multiplex ligation-dependent probe amplification) or a method based on analysis of NGS data (e.g., partial deletions of *MPV17* [76] and *POLG* [77].

## Variant interpretation

Nuclear variant interpretation and classification of pathogenicity should follow up-to-date guidelines, as presented by the American College of Medical Genetics and Genomics and the Association for Molecular Pathology (ACMG/AMP) [78], and relevant specifications by the Clinical Genomic Resource (ClinGen). The Association for Clinical Genomic Science (ACGS) has also evolved these guidelines providing clarifications and code strength modifications which undergo regular review. The latest version of the guidelines can be found in the Quality section on the ACGS website [79]. It is recommended that laboratories are stating on their reports which guidelines they are using for variant interpretation and classification.

Pathogenicity classification of mtDNA variants is often more challenging and the ClinGen expert panel has recently published specifications of the ACMG/AMP standards and guidelines for mitochondrial DNA interpretation [80]. In addition to these guidelines, a few brief key points are presented below to assist any laboratories querying the pathogenicity of mtDNA variants:

- Use of dedicated mtDNA databases: the MITOMAP database [81] represents the most comprehensive resource for mtDNA variation (listing both benign and pathogenic variants) and is updated regularly. The information provided includes locus, disease phenotype, GenBank frequency and a link to relevant publications.
- Frequency: Variant frequency must be considered when assessing pathogenicity and this should be interrogated for the general population. Variants with a frequency of ≥1/1000 curated GenBank sequences in the MITOMAP database or ≥1/1000 homoplasmic sequences in gnomAD v3.1 [82] and without any disease association are generally unlikely to be pathogenic.
- Haplogroups: mtDNA haplogroups define geographic origins of mtDNA variation accumulated during human evolution. MITOMASTER can be accessed through the MITOMAP website [81] and provides a prediction of the variant distribution in different haplogroups that can be considered in the evaluation of variant frequency.
- Heteroplasmy: The majority of pathogenic mtDNA variants are heteroplasmic. The variant level must be accurately determined and it is essential that this be interpreted in the context of the tissue tested. The variant level in a clinically affected tissue should correlate with the clinical presentation and any additional histochemical and/or biochemical findings.
- Homoplasmy: homoplasmic variants inherited from a homoplasmic clinically unaffected mother are unlikely to be pathogenic. However, there are known pathogenic homoplasmic variants which exhibit variable penetrance (e.g., *MT-RNR1* m.1555A>G, *MT-TI* m.4300A>G, *MT-ND4* m.11778G>A p.(Arg340His), other variants associated with LHON) and this must be considered.
- Evolutionary conservation and functionality: amino acid and nucleotide conservation between different species may be evaluated for protein-coding variation using in silico prediction tools. Special consideration should be given to mt-tRNA variants where the impact on the canonical structure and function should be assessed. Specialised databases for mt-tRNA variants may be consulted [83]. MitoTIP [84] provided through the MITOMAP database gives an in silico prediction score that may be used in addition to other evidence, for the assessment of mt-tRNA variants.
- Similar to the interpretation of nuclear variants, mtDNA variant evaluation should incorporate and consider all related family history and available clinical and histopathological findings. Laboratories may wish to seek further advice from specialised laboratories for interpretation of mtDNA variants.
- Muscle and other tissue biopsies may still be required to guide further functional evaluation of prioritised variants and provide evidence for establishing pathogenicity (see below).

Functional evaluation of both mtDNA and nuclear DNA variants can be extremely helpful in providing further evidence for or against pathogenicity. Muscle and/or other tissue biopsies may be required for these studies, which can be carried out by specialised laboratories. Illustrative examples include: single-fibre studies to investigate mtDNA variant segregation with a histochemical defect [85]; muscle histology and mtDNA deletion analysis to investigate pathogenicity of a nuclear variant detected in a gene associated with adult-onset mtDNA maintenance disorder; skin biopsy to measure PDH enzyme activity when a variant of uncertain significance is identified in a PDH deficiency associated gene.

It is essential to report the classification of the variant, i.e., pathogenic, likely pathogenic or uncertain significance. Variants classified as benign or likely benign are not routinely included in patient reports. It may not be appropriate to report variants of uncertain significance. This is dependent on local practice and whether further investigations/information may aid interpretation; further UK guidance is available from ACGS [17].

## Reporting

A molecular genetics report represents a lifelong document for the patient, their family and the referring clinician. It is therefore crucial that the information provided in the report is comprehensive, clear and succinct, and provides a full interpretation and unambiguous answer to the clinical question asked. In addition to outlining key limitations and stating the sensitivity and specificity, the report should contain full details of the regions analysed (e.g., specific exons, genes, panels) and the methodology used (e.g., kit references and versions used if applicable); alternatively, the report should include a statement that this information is available from the laboratory (or via a website). Guidance on general considerations for diagnostic molecular genetics reporting has been previously published [86, 87] and should be adhered to.

Variant nomenclature should follow current international guidelines as described by the Human Genome Variation Society (HGVS) [88] and gene naming convention should follow the approved HUGO Gene Nomenclature Committee [89]. Any reported coding variants should be described at the level of the nucleotide and the predicted protein change; this is applicable to both nuclear and mitochondrial DNA variants. The reference sequence used should also be provided.

In any diagnosis of mitochondrial disease (nuclear- or mtDNA-related), it is recommended that the report states any recurrence risk and implications to other family members and advises referral to clinical genetics or a specialist mitochondrial service for genetic counselling and appropriate family follow-up. In cases where no diagnosis has been reached (no variant detected or a variant of uncertain clinical significance that may warrant further investigation), the report should include a comment requesting additional clinical information or other samples for further appropriate testing (e.g., muscle biopsy); depending on the extent of testing undertaken, it may also be appropriate to comment on the potential to proceed to further testing/analysis (e.g., analysis of gene panels for clinically overlapping non-mitochondrial disorders, or gene-agnostic analysis). Special consideration should also be taken when reporting mono-allelic dominant SDHx variants that are associated with predisposition to cancer (pheochromocytoma and paraganglioma, reviewed in [90]; this risk should be included in the report and referral to clinical genetics recommended.

More specific considerations for reporting mtDNA variants are given below:

- The tissue of origin (e.g., blood, urine, buccal, muscle, etc.) should be stated in the report.
- The mitochondrial genome is conventionally numbered sequentially with reference to a whole mitochondrial genome reference sequence prefixed by ‘m.’. The appropriate reference sequence to use is currently GenBank NC_012920.1. This is a revised version of the Cambridge Reference Sequence [8] which maintains some historical nucleotide numbering (from the original Cambridge Reference Sequence) by indicating the absence of a nucleotide at m.3107 as ‘N’.
- For reportable mtDNA variants, the variant level, if heteroplasmic, or homoplasmy should be stated where possible.
- The limit of detection of the methodology used for mtDNA screening should be included.
- Interpretation of results should consider the age and sex of patient, sample type tested, limit of detection and sensitivity/specificity of the method used.
- Pathogenic and/or likely pathogenic mtDNA variants that are detected in a clinically affected patient at a mutant load that is consistent with the clinical phenotype, should be reported as confirming or consistent with a diagnosis of mitochondrial disease.
- If a (likely) pathogenic heteroplasmic mtDNA variant is detected at a lower mutant load in the tissue(s) tested than is likely to cause the patient’s symptoms, then this may be an incidental finding. Testing of additional tissues and advice from a specialist centre may be helpful. If it is concluded that the variant does not explain the phenotype, then it may be appropriate to report the variant due to reproductive implications and risks to family members; however, local policies on reporting of incidental findings should be followed.
- The report should include comment regarding any associated risk of developing (additional) symptoms, based on the available evidence for the particular pathogenic/likely pathogenic mtDNA variant. This is applicable to probands and their relatives whether affected or asymptomatic. For pathogenic mtDNA variants that are associated with skewed tissue segregation, additional samples may be requested to further refine risk when the initial analysis is carried out in blood-derived DNA. In addition, for certain variants (e.g., LHON primary pathogenic variants), the risk to extended family members is complicated by sex and age-dependent penetrance and this should be considered in the report.
- For female patients that harbour a (likely) pathogenic mtDNA variant and are/or will be of reproductive age, the report should include a statement that they are at risk of transmitting this variant to their offspring and reproductive advice is available from clinical genetics and/or a specialised mitochondrial service. In addition, testing can be offered to their maternal relatives as appropriate following genetic counselling.
- For male patients that harbour a (likely) pathogenic mtDNA variant, the report should include a statement that they are not at risk of transmitting the variant to their offspring. However, maternal relatives remain at risk and testing can be offered as appropriate following genetic counselling.
- When no pathogenic mtDNA variant is identified, the report should include a statement that this result decreases the likelihood of an underlying mtDNA genetic defect but does not fully exclude this.
- For familial testing when the variant is not detected, the report should include a statement that this result reduces but does not completely exclude the possibility that the familial variant may be present in other tissues not tested and/or at low levels that are below the limit of detection of the method used. Therefore, for females, where the familial variant is not detected, the risk of transmission to any offspring is reduced but not completely excluded.
- For prenatal reports, interpretation and conclusions should be made on the basis of available evidence for the given mtDNA variant including familial data. Further details are provided in the section ‘Reason for referral and genetic testing strategies—reproductive options and associated genetic testing’, although ideally these reports should be issued by a specialist centre. All prenatal reports should include the result of maternal cell contamination testing.

## Quality assurance

It is recommended that laboratories follow established good laboratory practices in accordance with guidelines for quality assurance in molecular genetic testing [91]. Furthermore, laboratories should aim to demonstrate compliance with internationally recognised standards (e.g., ISO standards 15189:2012 Medical laboratories) by achieving formal accreditation. Participation in an appropriate External Quality Assessment (EQA) scheme on an annual basis is encouraged, as it provides independent evidence of proficiency, facilitates ongoing quality assurance and competence, and promotes education. In addition, inter-laboratory exchange of samples may be arranged wherever it is not possible to participate in a formal EQA scheme.

## Discussion

Mitochondrial disease comprises a vast range of clinical phenotypes due to the dual involvement of the mitochondrial and nuclear genomes, which often makes clinical and molecular diagnosis complex and challenging. This is further confounded by the unique characteristics of mtDNA variants in terms of inheritance, heteroplasmy, and threshold effect. The recommendations presented here were ratified by the UK’s Association for Clinical Genomic Science following UK-wide laboratory consultation and constitute an update on previous guidance from the same group to include recent advances in mitochondrial genomic testing. The objective of this publication is to present these recommendations to an international scientific audience and highlight current genetic strategies for the molecular diagnosis of mitochondrial disease including aspects of testing, interpretation and reporting for patients and their families.

## Data access statement

No new data were created during this study.
